# Association between biliary complications and technique of hilar division (extrahepatic vs. intrahepatic) in major liver resections

**DOI:** 10.1186/1477-7819-4-59

**Published:** 2006-08-31

**Authors:** Vassileios Smyrniotis, Nikolaos Arkadopoulos, Kassiani Theodoraki, Dionysios Voros, Ioannis Vassiliou, Andreas Polydorou, Nikolaos Dafnios, Evangelos Gamaletsos, Kyriaki Daniilidou, Dimitrios Kannas

**Affiliations:** 1 Second Department of Surgery University of Athens School of Medicine, Aretaieion Hospital, Athens, Greece; 2 Pathology Laboratory, University of Athens School of Medicine, Aretaieion Hospital, Athens, Greece

## Abstract

**Background:**

Division of major vascular and biliary structures during major hepatectomies can be carried out either extrahepatically at the porta hepatic or intrahepatically during the parenchymal transection. In this retrospective study we test the hypothesis that the intrahepatic technique is associated with less early biliary complications.

**Methods:**

150 patients who underwent major hepatectomies were retrospectively allocated into an intrahepatic group (n = 100) and an extrahepatic group (n = 50) based on the technique of hilar division. The two groups were operated by two different surgical teams, each one favoring one of the two approaches for hilar dissection. Operative data (warm ischemic time, operative time, blood loss), biliary complications, morbidity and mortality rates were analyzed.

**Results:**

In extrahepatic patients, operative time was longer (245 ± 50 vs 214 ± 38 min, p < 0.05) while the overall complication rate (55% vs 52%), hospital stay (13 ± 7 vs 12 ± 4 days), bile leak rate (22% vs 20%) and mortality (2% vs 2%) were similar compared to intrahepatic patients. However, most (57%) bile leaks in extrahepatic patients were grade II (leaks that required non-operative interventional treatment, while most (70%) leaks in the intrahepatic group were grade I (leaks that resolved and presented two injuries (4%) of the remaining bile ducts (p < 0.05).

**Conclusion:**

Intrahepatic hilar division is as safe as extrahepatic hilar division in terms of intraoperative blood requirements, morbidity and mortality. The extrahepatic technique is associated with more severe bile leaks and biliary injuries.

## Background

Hilar division is a critical step in major liver resections. The technique used should secure integrity of the bile ducts as well as unhindered bile flow and uninterrupted blood flow of the liver remnant [1-7]. Extrahepatic hilar dissection has been established as a standard process during major liver resections as it reduces blood loss by dividing the vascular structures of each portal pedicle before the parenchymal liver transection. This process involves dissection of the fibrous sheath that envelops the portal triad and individual division of the vascular and biliary tributaries. However, hilar dissection may be extremely difficult in cases of extensive scarring due to previous surgery and may be complicated by accidental injuries of the vascular and biliary structures that are vital for the liver remnant [6-9]. Intrahepatic division of the vascular and biliary ramifications during the parenchymal liver transection has emerged as a safer alternative, since the dissection plane stays far away from the bifurcation of the portal triad. Although this technique appears to lessen the likelihood of damage to the structures of the porta hepatic, it is more often complicated by intraoperative bleeding during parenchymal transection since the blood flow remains uninterrupted [6,7,9].

The scarcity of studies comparing the two techniques prompted us to carry out the present retrospective analysis. We hypothesized that compared to extrahepatic dissection, intrahepatic hilar division may be associated with less biliary complications and especially less injuries to the biliary structures of the liver remnant.

## Methods

In the last five years (2000–2005), in our institution, one hundred and fifty patients were subjected to major hepatectomy, i.e. resection of more than 3 segments (Couinaud's classification). According to the type of portal triad division, all patients were retrospectively allocated into two groups. Fifty patients that underwent extrahepatic hilar division were assigned as extrahepatic group (EHD group, n = 50) and one hundred patients that underwent intrahepatic portal pedicle division were assigned as intrahepatic group (IHD group, n = 100). Each group was operated by a dedicated surgical team directed by either author V.S. or author D.V. Each surgical team applied its preferred hilar division technique, either IHD or EHD. Patient demographics, preoperative liver function tests and indications for surgery are listed in Table 1.

**Table 1:** Preoperative characteristics of the patients subjected to liver resection with Intrahepatic (IHD) and Extrahepatic (EHD) hilar division.

	**IHD group (n = 100)**	**EHD group (n = 50)**
**1. Age (years) **(mean ± SD)	63 ± 11	63 ± 9
**2. Male(M) to female(F) ratio**	3/1 (75M/25F patients)	3/1 (38M/12F patients)
**3. Preoperative lab values (mean ± SD)**		
a. platelets × 10^12 ^(cell/L)	210.5 ± 91	215 ± 88
b. prothrombin time (INR)	0.98 ± 0.09	0.91 ± 0.08
c. bilirubin (μmol/L)	9.2 ± 4.1	9.5 ± 4.2
d. AST (U/L)	35 ± 15	36 ± 14
e. albumin (g/dl)	4.8 ± 0.7	4.6 ± 0.8
**4. Indication for resection**		
a. hepatocellular carcinoma (70)	46 (46%)	24 (48%)
b. metastatic carcinoma (60)	45 (45%)	15 (30%)
c. benign lesions (20)	9 (9%)	11 (22%)

**Table 2:** Operative characteristics of patients subjected to liver resection with Intrahepatic (IHD) and Extrahepatic (EHD) hilar division.

	**IHD group (n = 100)**	**EHD group (n = 50)**
**1. Type of liver resection n°(%):**		
a. right hepatectomy	60 (60%)	30 (60%)
b. left hepatectomy	20 (20%)	12 (24%)
c. extended right hepatectomy	12 (12%)	5 (10%)
d. extended left hepatectomy	8 (8%)	3 (6%)
**2. Parenchymal transection technique (%):**		
a. clamp crushing	18 (18%)	14 (28%) (p < 0.05)
b. sharp transection	82 (82%)	36 (72%)
**3. Vascular control technique**		
a.TVC*	6 (6%)	3 (6%)
b. Pringle maneuver	23 (23%)	10 (20%)
c. SHVE^+^	71(71%)	29(58%)
d. without vascular control	0 (0%)	8 (16%) (p < 0,05)
**4. Operative time **(minutes, mean ± SD)	214 ± 38	245 ± 50 (p < 0.05)
**5. Ischemic time **(minutes, mean ± SD)	40 ± 6	38 ± 8
**6. Blood loss **(ml, median-range)	540 (150–2600)	680 (150–3000)
**6. Ischemic preconditioning no (%)**	65(59%)	25(50%)

* TVC: Total Vascular Control

+ SHVE: Selective Hepatic Vascular Exclusion

**Table 3:** Complications following liver resection with Intrahepatic (IHD) and Extrahepatic (EHD) hilar division.

**Complications**	**IHD group (n = 100)**	**EHD group (n = 50)**
**Wound infection**	2	1
**Chest infection**	18	5
**Pleural effusion**	6	5
**Subphrenic abscess**	2	1
**Bile leakage**	20	11
**Bleeding (postoperative period)**	2	1
**Liver failure**	5	2
**ICU stay **(days, mean ± SD)	1 ± 0.5	1 ± 0.6
**Hospital stay **(days, mean ± SD)	12 ± 4	13 ± 7
**Hospital deaths (%)**	2(2%)	1(2%)

### Surgical techniques

The abdomen was entered in all patients by a bilateral subcostal incision. Following liver mobilization, intraoperative ultrasonography was used in all cases in order to determine tumor resectability and define the resection plane. In patients who underwent intrahepatic hilar division, the ramifications of the portal vein, hepatic artery and biliary ducts were transected as they were met during parenchymal transaction. Vascular control was achieved with either total vascular exclusion (TVE), Pringle maneuver or selective hepatic vascular exclusion (SHVE) in six, twenty-three and seventy-one patients respectively. Technical aspects of vascular control and liver transection have been described in detail in our previous reports [10,11]. TVE was achieved by simultaneous clamping of the hepatoduodenal ligament and the suprahepatic and infrahepatic inferior vena cava (IVC). Pringle's maneuver was carried out by applying a Satinsky clamp to the hepatoduodenal ligament and SHVE was accomplished by disconnecting the liver from the IVC and selective clamping of liver inflow and outflow (hepatic veins) without disturbing the flow in the IVC. The liver parenchyma was transected with Kelly clamps (crush clamping technique) or by sharp transection with a knife [10].

In patients who underwent extrahepatic hilar division, the portal vein, hepatic artery and biliary ducts were individually dissected in the hilum by opening of the peritoneal fascia. The portal vein and the hepatic artery branch were ligated and transected at the porta hepatic while the hepatic ducts were divided inside the liver. Afterwards, the liver parenchyma was transected either by the crush clamping technique or by sharp transection with the aid of TVC, Pringle's maneuver and SHVE in three, ten and twenty-nine patients respectively, while in eight patients the resection was accomplished without any type of vascular control. Following completion of the hepatectomy a drain was placed close to the liver cut surface in all patients of both groups.

In 59% of IHD and 50% of EHD patients we employed ischemic preconditioning, i.e. 10 min of ischemia (Pringle's maneuver) followed by 15 min of reperfusion, before the onset of vascular exclusion.

The two groups were compared in a retrospective manner. Operative time, warm ischemic time and blood loss were recorded for all patients. Furthermore, postoperative liver function was assessed by daily measurements of liver function tests [aspartate aminotransferase (AST), bilirubin, and prothrombin time (PT)]. Postoperative biliary complications, morbidity and mortality were evaluated and compared between the two groups. Complications included into the present analysis were those that required some kind of treatment and protracted the patients' hospital stay or resulted in death.

Biliary complications were further classified as: a. Bile leakage and b. Injury of the contralateral biliary ducts. The severity of bile leakage was graded as follows: grade I when draining was required for more than 10 days but leakage finally resolved and grade II when draining was complemented by a non operative intervention (CT-guided aspiration and/or stenting of the bile duct solved the problem) and grade III when reoperation was needed. Bile leakage was defined as the presence of any amount of bile in the drainage fluid or in intra-abdominal collections (bilirubin levels at least three times higher than serum levels) for more than 2 days postoperatively. Liver failure was determined as: bilirubin ≥ preoperative levels × 10, lasting for more than three days unrelated to biliary obstruction leak and/or INR≥ preoperative levels × 2 for more than 2 days after resection and/or significant ascites/encephalopathy.

The results are expressed as means ± SD (standard deviation). Statistical analysis was performed by the Mann-Whitney U test while the chi square test was used for categorical data as appropriate. P < 0.05 was defined as statistically significant. Calculations were conducted with the assistance of SPSS software (Chicago Ill, USA).

## Results

**Figure 1:**
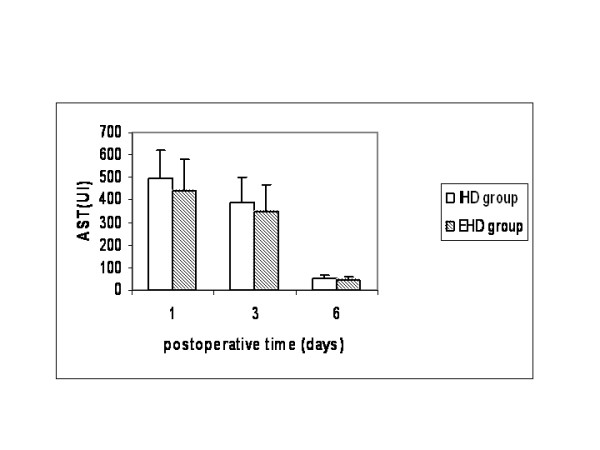
Plasma levels of aspartate aminotransferase (AST) in patients that underwent liver resectionwith intrahepatic hilar division (IHD group) and extrahepatic hilar division (EHD group).

**Figure 2:**
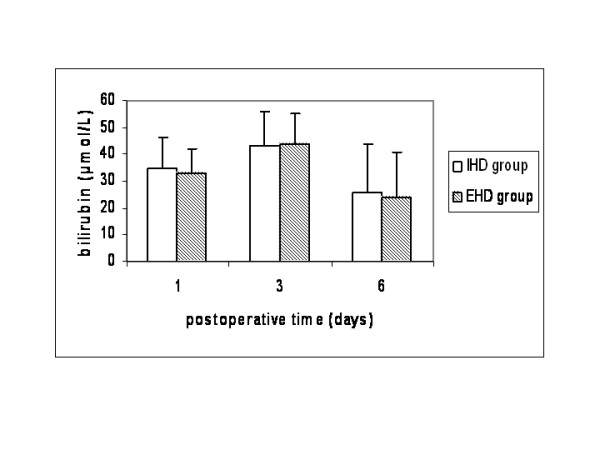
Plasma levels of bilirubin in patients that underwent liver resection with intrahepatic hilar division (IHD group) and extrahepatic hilar division (EHD group).

**Figure 3:**
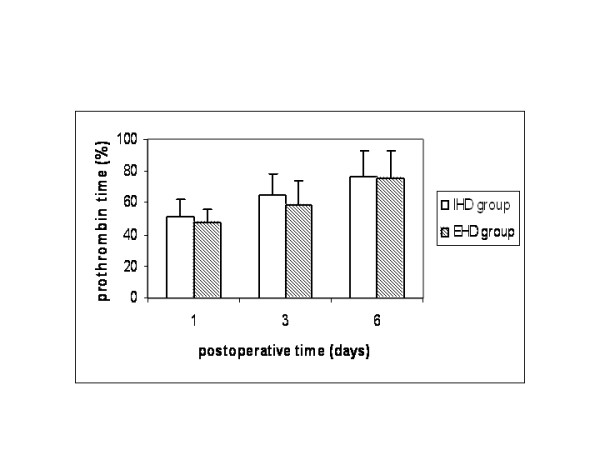
Prothrombin time in patients that underwent liver resection with intrahepatic hilar division (IHD group) and extrahepatic hilar division (EHD group).

Both groups were comparable regarding age, gender, preoperative liver function, comorbid diseases and indications for liver resection (Table 1). No patients had cirrhosis Child≥B or portal hypertension. Operative characteristics that might have an implication on patients' morbidity and mortality such as volume of resected liver, technique of parenchymal transection and type of vascular control were equally distributed between the two groups, except for the fact that more patients in the extrahepatic group (16% vs 0%) were operated on without any type of vascular exclusion (Table 2). Warm ischemic time was similar in both groups (40 ± 6 vs 38 ± 8 min), but operative time was longer in the extrahepatic group compared to the intrahepatic group (245 ± 50 vs 214 ± 38 min, p < 0.05). Liver function was similar between the two groups (Figures 1, 2, 3). Liver failure was observed in five patients in the intrahepatic group and two in the extrahepatic group.

No significant difference was demonstrated between the EHD and IHD group regarding ICU stay (1 ± 0.6 vs 1 ± 0.5 days), hospital stay (13 ± 7 vs 12 ± 4 days) and overall complication rate (55% vs 52%) (Table 3). Two patients (2%) died in the IHD group. The first was a patient in liver failure that developed hepatorenal syndrome six weeks later and the second a patient presenting with alcoholic cardiomyopathy who developed a fatal cardiac arrhythmia one week later. One patient (2%) in the EHD group had postoperative bleeding. The patient was reoperated on and hemorrhage was controlled by packing. Unfortunately he developed overwhelming sepsis and died two weeks later.

Bile leak that fit the criteria of our definition occurred in 20 patients in the IHD group (20%) and 11 patients in the EHD group (22%). Analysis of biliary complications in relation to their severity revealed that most leaks in the IHD group were grade I (14 patients or 70% of the leaks) while most leaks in the EHD group were grade II (6 patients or 55% of leaks). The difference in the distribution of the two grades of bile leaks in each group was statistically significant (p < 0.05). All grade II leaks were treated with CT guided aspiration and/or common bile duct stenting. No grade III bile leaks were recorded in this study.

Intraoperative biliary trauma occurred in two patients (4%) in the EHD group: one required a hepaticojejunostomy with the left hepatic duct and the other one suturing of a tear of the common hepatic duct over a T-tube. Both patients had an uneventful recovery.

Our statistical analysis did not show any of the other technical parameters (method of vascular control, method of parenchymal division) to have an influence on biliary complications.

## Discussion

Bleeding, biliary complications and the functional capacity of the liver remnant are major determinants of patients' outcome following major liver resections [4-7]. Intraoperative bleeding has been substantially reduced due to refinements and advances in the techniques of vascular control, parenchymal transaction and hemostasis. Nowadays, the majority of resections can be accomplished safely without blood transfusion [11-14]. Various methods have been proposed to preserve the functional capacity of the liver remnant [15,16]. Ischemia-reperfusion (I/R) injuries can be ameliorated considerably by pharmaceutical or mechanical preconditioning and intermittent vascular control [15,16]. The volume of the liver remnant, when anticipated to be less than 20% of the standard volume, can be enhanced by occluding the branch of the portal vein to the tumor-bearing lobe. Preoperative redirection of the whole portal flow to the healthy liver augments regeneration by 30 to 70% within 3–4 weeks and significantly reduces complications and liver failure [17-20].

Epidemiological and procedural prognostic factors in liver resections have been extensively evaluated in large retrospective analyses. Jarnagin et al demonstrated that the extent of liver resection and blood loss were the only independent factors for perioperative morbidity and mortality [5]. Belghiti et al claimed that a concomitant extrahepatic procedure was the only independent predictor of operative death in patients without underlying liver disease [15]. Vauthey et al found that combining an extrahepatic procedure with an extended liver resection reduces the risk [13]. Imamura et al stated that liver resections can be carried out without mortality in high volume medical centers by well trained hepatobiliary surgeons who take into account the balance of liver functional reserve and the volume to be resected [21].

Biliary complications constitute a significant portion of post-hepatectomy morbidity and mortality [6-9]. It appears that patients with extended liver resections or biliary malignancies that necessitate hepaticojejunostomies are more prone to develop liver or biliary complications [9,22]. Devascularization of the biliary ducts during hilar dissection should be considered the main factor compromising their anatomical and functional integrity and predisposing to sepsis and liver failure [16,21]. Lam et al found that 23 to 38% of postoperative deaths were attributed to biliary system failure [23]. The same authors recommended avoiding extensive dissection of the hilar area that may cause injury of the hepatic ducts resulting in bile leakage, biliary stricture or obstruction. Similar findings have been reported by Lo et al and Tanaka et al, who emphasized that reoperation for biliary complications carries a mortality rate as high as 70% [7,22]. Fortunately, the majority of biliary complications, as in our study, were amenable to nonsurgical treatment [21,22]. Launois et al [24] standardized the Glissonian approach, stapling en mass the portal pedicle in order to avoid hilar dissection, a technique that has yielded very good results in a number of studies [12,14]. However, accidental stapling of the opposite portal pedicle has been reported and the technique should be avoided when tumors are close to the hilar region [12,14].

Intrahepatic division of the structures of the portal pedicle as they are met during the transection of the liver looks like a reasonable alternative as it obviates the dissection of the hilar region and spares the vital structures of the liver remnant from accidental injury [7,12]. However, the technique may jeopardize the optimum tumor free margin in centrally located malignancies and may be complicated by bleeding, if employed without vascular control [1,5,13,25].

Our study demonstrated that intrahepatic division of the porta hepatis shortens overall operative time [214 ± 38 vs 245 ± 50 min] without affecting the warm ischemic time. The higher rate of blood loss in the extrahepatic group should be attributed to the fact that 16% of the patients were operated on without vascular control. However, it should be emphasized that no difference was detected when vascular control was applied. It appears that vascular control during intrahepatic liver dissection reduces blood loss equally to extrahepatic hilar division when vascular ligation precedes liver transection. Morbidity and mortality were similar in both groups and all three deaths were unrelated to the technique of hilar division. The liver failures recorded in this study were probably due to either extended resection, fatty liver or preoperative chemotherapy.

Our findings regarding complications and mortality rates are in accordance with those mentioned in the literature [1,6,7,23,26]. Regarding biliary leaks, most studies in the literature cite rates in the range of 7–10% [21-23,26]. The higher rate recorded in the present study (20–22%) can be attributed to our very low threshold for the definition of leak. However, by analyzing the biliary complications in relation to their severity we demonstrated that dissection of the hilar region in order to identify the vascular and biliary structures is associated with more severe complications as reflected by the higher rate of grade II bile leaks in the EHD group compared to the IHD group (57% vs 30%) and the two accidental injuries to the hepatic ducts that occurred in EHD patients and required either a hepaticojejunostomy or suturing over a T-tube. Even though we, as many other surgeons who employ extrahepatic dissection divide the hepatic duct inside the liver, it is the process of dissection at the porta hepatis in order to recognize and isolate the vascular structures that can cause accidental injury to the biliary tree.

Injury of the biliary ducts of the liver remnant is usually manifested as bile leakage, stricture or obstruction and should be considered a potentially lethal complication due to the risk of sepsis and liver failure [7,9,19,21,22]. Bile leaks from the cut surface are usually self-limited when they originate from unstiched subsegmental branches [19,22]. By contrast, leaks from segmental bile ducts or the stump of the transected hepatic duct are difficult to heal and may require endoscopic stenting or reoperation. Aberrations of the biliary system are not infrequent (20–30%) and the surgeon we should be aware of the fact that segments of one lobe can drain into the opposite lobe, close to the bifurcation of the common hepatic duct [15]. In such cases, intrahepatic hilar division lessens the possibility of unwanted transection of bile ducts that drain segments of the liver remnant. Preoperative imaging of the biliary tree may be useful in discovering such biliary aberrations and planning the operative strategy [15,24,26]. However, the cost-effectiveness of routine preoperative biliary imaging needs to be further investigated in clinical trials.

In conclusion, intrahepatic division of the porta hepatis in major liver resections is similar to extrahepatic division, in terms of blood loss and warm ischemic time while at the same time it expedites overall operative time. Morbidity and mortality are similar between the two techniques, but biliary complications are more severe in patients undergoing extrahepatic division of the portal pedicle. In these patients accidental injury of the contralateral biliary ducts may also occur. As a result, we tend to favor the intrahepatic division of the porta hepatis under vascular control, for most liver resections. However, when tumors are close to the hilar region, extrahepatic division should be undertaken to optimize the tumor-free margin. Indications for the application of each approach should be further clarified by a prospective randomized trial.

## Competing interests

The author(s) declare that they have no competing interests.

## Authors' contributions

VS, NA, DV, JV, EG, AP: acquisition of data, drafting of the manuscript

VS, NA, DK: Study design

VS, KT, ND, KD: Analysis and interpretation of data

All authors read and approved the final manuscript
